# Assessing laboratory animal welfare: the crucial importance of construct validity

**DOI:** 10.1177/00236772251380871

**Published:** 2026-03-09

**Authors:** Georgia Mason

**Affiliations:** Campbell Centre for the Study of Animal Welfare, University of Guelph, Guelph, ON, Canada

**Keywords:** Animal use, ethics and welfare, distress, laboratory animal welfare, public policy, refinement

## Abstract

Assessing laboratory animals’ welfare – their current and/or past subjective affective states – is essential for ethical and regulatory reasons (and central to biomedical research into, for example, pain, nausea or anxiety). But this is challenging; and in the quest for quantification (and perhaps simplicity), it can be tempting to overlook construct validity. Nevertheless, that our indicators have good construct validity – that is, they accurately reflect the construct or concept of interest – is essential. This is true whether we are interested in short-term emotions like fear, longer-term mood-like states such as malaise, or markers of cumulative stress over a project or even a lifespan. Without it, welfare assessments risk being incorrect: inaccurate and unhelpful for the animals they aim to evaluate and assist. Here (summarising text from a forthcoming edited book), I introduce five validatory tests, as well as highlighting the importance of considering indicators’ responsiveness/sensitivity and selectivity/specificity. I also outline how these principles could help improve the construct validation of both humane endpoints and retrospective severity assessments. Careful construct validation can never fully solve the ‘Other Minds’ problem: that animals’ subjective experiences are private (such that we can never measure them, only infer them). However, done well, construct validation would add additional logical rigour to laboratory animal welfare assessment, increase its accuracy, and make benchmarking (e.g. severity classification) more transparent.

## Évaluation du bien-être des animaux de laboratoire : importance cruciale de la validité de construction

Résumé

L’évaluation du bien-être des animaux de laboratoire - leurs états affectifs subjectifs actuels et/ou passés - est essentielle pour des raisons éthiques et réglementaires (et centrale pour la recherche biomédicale sur la douleur, les nausées ou l’anxiété, par exemple). Elle s’avère toutefois difficile à faire, et dans la quête de quantification (et peut-être aussi de simplicité), il peut être tentant de négliger la validité de construction. Il est cependant vrai qu’il est essentiel que nos indicateurs aient une bonne validité de construction – c’est-à-dire qu’ils reflètent fidèlement la construction ou le concept d’intérêt. Que nous soyons intéressés par des émotions à court terme comme la peur, des états d’humeur à plus long terme comme le malaise, ou par des marqueurs de stress cumulatif sur un projet ou même la durée d’une vie, le fait. Sans les évaluations du bien-être risquent d’être incorrectes : inexactes, et donc inutiles pour les animaux qu’elles visent à évaluer et à aider. Je présente ici cinq tests de validation (résumant le texte d’un prochain livre édité), ainsi que l’importance de considérer la réactivité/sensibilité et la sélectivité/spécificité des indicateurs. J’explique également comment ces principes pourraient aider à améliorer la validation de construction des critères d’intervention humains et des évaluations rétrospectives de la gravité. La validation minutieuse des constructions ne peut jamais résoudre complètement le problème des « autres esprits » : les expériences subjectives des animaux sont privées (de sorte que nous ne pouvons jamais les mesurer, mais seulement les inférer). Si elle est bien réalisée, elle ajouterait cependant davantage de rigueur logique à l’évaluation du bien-être des animaux de laboratoire, augmenterait sa précision et rendrait l’étalonnage (par exemple, la classification de la gravité) plus transparent.

## Bewertung des Wohlergehens von Labortieren: Die entscheidende Bedeutung der Konstruktvalidität

Die Beurteilung des Wohlergehens von Labortieren – ihrer aktuellen und/oder vergangenen subjektiven Gefühlszustände – ist aus ethischen und regulatorischen Gründen unerlässlich (und zudem von zentraler Bedeutung für die biomedizinische Forschung, z. B. zu Schmerzen, Übelkeit oder Angstzuständen). Sie ist allerdings eine Herausforderung, und beim Streben nach Quantifizierung (sowie möglicherweise auch nach Einfachheit) kann die Konstruktvalidität leicht ignoriert werden. Es ist jedoch unerlässlich, dass unsere Indikatoren eine gute Konstruktvalidität aufweisen, d. h. dass sie das betreffende Konstrukt oder Konzept genau widerspiegeln. Dies gilt unabhängig davon, ob wir uns für vorübergehende Emotionen wie Angst, länger anhaltende Stimmungszustände wie Unwohlsein oder Marker für kumulativen Stress während eines Projekts oder sogar während der gesamten Lebensdauer interessieren. Ohne sie besteht die Gefahr einer falschen Bewertung des Wohlergehens, d. h. einer Bewertung, die ungenau und somit für die Tiere, die damit beurteilt und unterstützt werden sollen, nicht hilfreich ist. Hier stelle ich (zusammenfassend aus einem in Kürze erscheinenden Sammelband) fünf Validierungstests vor und unterstreiche zudem die Bedeutung der Berücksichtigung der Reaktionsfähigkeit/Sensitivität und Selektivität/Spezifität von Indikatoren. Außerdem lege ich dar, wie diese Prinzipien dazu beitragen könnten, die Konstruktvalidität sowohl von humanen Endpunkten als auch von retrospektiven Schweregradbewertungen zu verbessern. Eine sorgfältige Konstruktvalidität kann das Problem der „Other Minds” niemals vollständig lösen: Die subjektiven Erfahrungen von Tieren sind privat (wir können sie niemals messen, sondern nur ableiten). Bei korrekter Durchführung würde sie jedoch die Bewertung des Wohlergehens von Labortieren logisch stringenter machen, ihre Genauigkeit erhöhen und Benchmarking (z. B. Schweregradklassifizierung) transparenter gestalten.

## Evaluación del bienestar de los animales de laboratorio: la importancia crucial de la validez de constructo

Resumen

Evaluar el bienestar de los animales de laboratorio –sus estados afectivos subjetivos actuales y/o pasados– es esencial por motivos éticos y normativos (además de fundamental para la investigación biomédica sobre, por ejemplo, el dolor, las náuseas o la ansiedad). Pero esto es un reto; y en la búsqueda de la cuantificación (y quizás de la simplicidad), puede resultar tentador pasar por alto la validez de constructo. No obstante, es esencial que nuestros indicadores tengan una buena validez de constructo; es decir, que reflejen con precisión el constructo o concepto de interés. Esto es cierto tanto si nos interesan emociones a corto plazo como el miedo, estados más duraderos similares al estado de ánimo como el malestar, o indicadores de estrés acumulado a lo largo de un proyecto o incluso de un ciclo vital. Sin esto, las evaluaciones del bienestar corren el riesgo de ser incorrectas: inexactas y, por tanto, poco útiles para los animales a los que pretenden evaluar y asistir. Aquí (resumiendo el texto de un libro de próxima publicación), presento cinco pruebas de validación, además de resaltar la importancia de considerar la capacidad de respuesta/sensibilidad y la selectividad/especificidad de los indicadores. Asimismo, expongo cómo estos principios podrían contribuir a mejorar la validación de constructo tanto de los puntos finales humanitarios como de las evaluaciones retrospectivas de severidad. Una cuidadosa validación de los constructos nunca podrá resolver por completo el problema de las «Otras Mentes»: que las experiencias subjetivas de los animales son privadas (de modo que nunca podemos medirlas directamente, solo inferirlas). Sin embargo, si se hace bien, añadiría más rigor lógico a la evaluación del bienestar de los animales de laboratorio, aumentaría su precisión y haría más transparente la evaluación comparativa (por ejemplo, la clasificación de la gravedad).

Assessing laboratory animals’ welfare – their current and/or past subjective affective states (Table 1) – is essential for ethical and regulatory reasons (and central to biomedical research into, for example, pain, nausea or anxiety).^1–3^ This is challenging, however. Animals cannot tell us how they are feeling, and relevant indicators – measurable variables from which to infer non-measurable feelings – can vary across states (Table 1), as well as across species, strains, age classes and welfare challenges (e.g. specific diseases being modelled).^4-7^ Consequently, any method yielding seemingly objective values with apparent ease can be appealing (be this quick cage-side checks, simple behavioural tests, automated readouts from sensors, or impressive-looking ‘composite scores’). But in the quest for quantification (and perhaps simplicity of assessment), *construct validity* must not be overlooked.

**Table 1.** Negative affective states and related constructs relevant to laboratory animal welfare.

Negative affective state (or related construct): definitions and examples^8^			*Potential indicators of presence/intensity* ^*8*^
*Affective state*	*Definition*	*Examples (as reported by humans, and inferred in other species)*	
Emotions (including homeostatic/primordial emotions induced by homeostatic needs, sickness or injury)	Acute states closely tied to specific rewarding (preferred) or punishing (aversive) external or internal events	• Fear • Pain • Hunger • Nausea • Thirst	• Avoidance/escape • Changes in heart rate • Vocalisations • Facial expressions • Postures
Moods (including long-term states induced by sickness or injury)	Longer-term states that, for human subjects, may last from hours to weeks, feel as if they have no obvious immediate cause, and change the readiness with which subjects experience positive or negative emotions	• Malaise • Anxious moods • Depressed moods	• Judgement biases • Propensity to freeze/startle • Reactivity to noxious stimuli • Apathy • Changes in coat/body condition • Susceptibility to disease, both infectious and non-infectious
Affective disorders	Prolonged, disproportionately negative affective states that are hard to reverse	• Generalized anxiety disorder • Post-traumatic stress disorder • Major depressive disorder	• As for moods, but more severe • Anhedonia • Abnormal repetitive behaviours
Temperament (affective personalities)	Biological predispositions to exhibit particular affective states: traits, potentially stable over the lifetime	• Genetic, developmental or lesion-induced models of anxiety, depression or hyperalgesia	• As for moods or affective disorders, but stable over lifespans
Cumulative affective experience, or cumulative adversity	The summed experience of emotions, moods and affective disorders across a prolonged period, e.g. a lifespan	• Poor/good quality of life • A life (not) worth living • Cumulative suffering or severity	• As for affective disorders • Hippocampal volume loss • Premature senescence • Physiological markers of cumulative ‘wear and tear’ including high allostatic load (a composite of stress-/ageing-sensitive measures) and shortened telomeres

Construct validity means how accurately a metric reflects a construct or concept of interest (such as how well a cognitive test score reflects intelligence, for instance).^9,10^ When assessing animal welfare – regardless of whether our construct of interest is, say, fear, contentment, or quality of life – the indicators we use must thus reflect ‘*“ground-truth”* – the state that the animal is actually in’,^11^ yielding ‘numbers that really do reflect the welfare as experienced by the animals’.^12^ Significant consequences can result if they do not. Using affect indicators of questionable validity may, for instance, help explain why few new therapies for humans have emerged from animal-based biomedical research on pain^13–15^ and depression.^16–18^ Using affect indicators of questionable validity also risks welfare assessments being incorrect and unhelpful for animals.^10,19^ Construct validation is thus essential for ensuring that metrics mean what we hope they do. Happily, validatory methods are described in affect-focused biomedical studies, animal welfare research, veterinary research, and psychological research on human and animal emotions.^10^ Five key methods emerge in these literatures: validation tests that potential indicators should pass (cf. the negative state indicators in Table 2).

**Table 2.** Five tests for the construct validation of indicators of negative affect (poor welfare), with some laboratory animal-relevant examples. Each test relies on different assumptions, and so indicators can be used with greater confidence the more tests they pass.

Test	Methodology	Key underlying assumptions	Relevant examples
1. Using humans as models for other species	Assess measurable signs in humans self-reporting the negative state of interest	Biological homology between humans and other species	Validating some indicators of canine nausea (e.g. hyper-salivation, groaning, high plasma levels of Substance P), by studying correlates of human nausea^20^
2. Exposure to known or aversive stimuli	Assess measurable signs in animals exposed to stimuli or contexts they avoid if given a choice	Avoidance (e.g. struggling, flight, learned aversions, learned escape responses) reflects negative affect	Validating immobility and thigmotaxis as negative affect indicators in zebrafish, by increases in these behaviours in tank designs this species finds aversive^21^
3. Exposure to fitness-threatening stimuli	Assess measurable signs in animals exposed to circumstances that would threaten their (ancestors’) fitness in the wild	Animals evolved to feel negative affect in response to threatened fitness; our subjects’ brains retain this legacy today	Validating stereotypic bar-mouthing as a welfare indicator in mice, by its elevation after premature loss of the mother^22^
4. Pharmacological validation	Assess measurable signs in animals given affect-modulating drugs	The drugs influence affective states in the ways that we think they do	Validating Elevated Plus Maze behaviour (time in the closed arms ) as evidence of anxiety, by its reduction by anxiolytic drugs^23^
5. Validation by other indicators	Assessing whether novel measurable signs covary with indicators of affect already validated in our species	The indicators relied upon are validated	Validating ‘grimace’ scales for scoring pain, by their correlation with prevalidated indicators (e.g. tooth-grinding, lameness, inactive hunched postures)^24,25^

Along with passing validatory tests, ideal indicators should be highly responsive, sensitively reacting to all relevant changes in affect (even subtle ones) in an incremental manner.^9^ They should also be highly selective, only reflecting the specific affective states we wish to assess. Sadly, perfectly ideal indicators do not exist. But understanding the properties of those we have can help identify the best metric (or combination) for a given task. As Dawkins^26^ put it, this is like ‘Be[ing] aware of the limitations of your materials before you start building a house’. To illustrate, weight loss can be validly used to infer suffering in clinical models, but only if we appreciate *a priori* when it can be insensitive (e.g. in acute conditions where animals rapidly become moribund,^27^ or where effects are masked by reduced activity or even changes that increase bodyweight like elevated corticosteroid levels^28^ or ascites^29^; see also Talbot et al.^5^). Such understanding can in turn reduce errors: failing to detect changes in affective states that are present (a.k.a. false negatives/false null conclusions), or mistakenly inferring changes in affective states that are *not* present (a.k.a. false positives/false leads).

**Figure 1.**
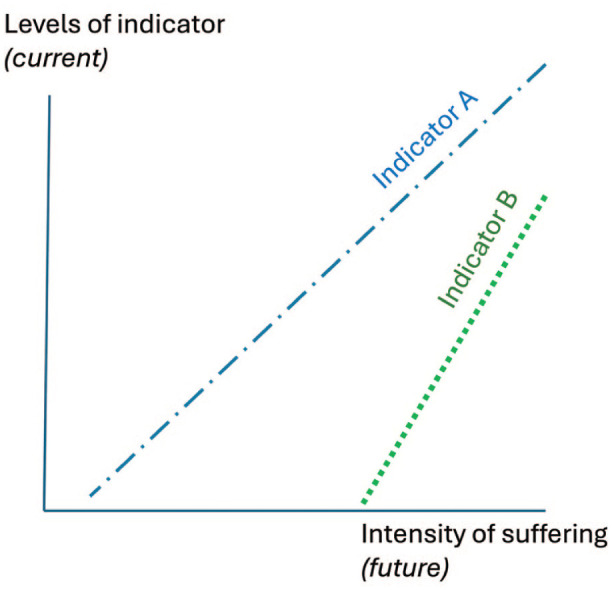
Two (imaginary) indicators that could be used to identify future suffering and consequent endpoints. Both indicators covary with future suffering, anticipating it in a graded, incremental way. However, Indicator A is potentially more useful for truly humane endpoints than Indicator B because it responds to lower levels of predicted future suffering, so allowing earlier intervention.

Formal principles of construct validation could help improve humane endpoints in terms of both their validity and their humaneness. To serve as accurate proxies (e.g. triggering a study’s end or a subject’s removal), humane endpoints should statistically predict severe suffering. Studies developing these essentially use Test 5 (see Table 1 and Figure 1): data collected from animals subsequently assessed for clinical scores warranting euthanasia are retrospectively analysed to identify which potential indicators differentiate between subjects who will live or die (and perhaps also between experimental animals and healthy controls).^4–6,27,30^ However, such studies are rare; they assume that clinical scores are valid; and, furthermore, the resulting ‘humane’ endpoints may still involve much suffering^6,30^ (see also Figure 1). Together, this makes further research into humane endpoints essential, and meeting this need should arguably involve new, complementary validatory tests. For example, for animal models of disease, this could involve liaising with patient groups, doctors and human clinical researchers to identify measurable signs that precede severe suffering – even desires for medically assisted dying – in relevant human patients (cf. Test 1 in Table 2).

**Figure 2.**
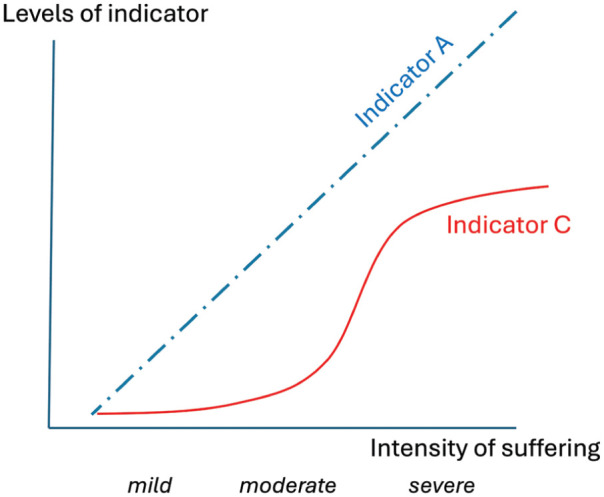
Two (imaginary) indicators useful for assessing severity in different ways. Indicator A is responsive in an incremental way across the whole range of negative affect (as in Figure 1), and so would be ideal for experimental research comparing refinements. In contrast, Indicator C, with its threshold and ceiling effects, cannot differentiate between degrees of mild, moderate or severe negative affect within each category. Nevertheless, Indicator C would be very useful in severity banding (e.g. for identifying the threshold between moderate and severe states).

Such principles should also inform severity assessment: an even more challenging task because indicators must reflect not just relatively more or less suffering (as in Figure 1), but particular levels (and ideally even their boundaries: see Figure 2). Directive 2010/63/EU,^31^ for example, defines ‘mild’ as causing only short-term mild pain, suffering or distress (i.e. mild negative emotions cf. Table 1); ‘moderate’ as causing moderately negative emotions and/or longer-term negative states (e.g. negative moods, Table 1) that are only mild or only moderately impair overall condition (presumably via their cumulative impact); and ‘severe’ as causing severely negative emotions, and/or negative moods that are moderately to highly negative or severely impair overall condition. The Directive also lists types of procedure judged to fall within each category. However, it does not supply evidence for these judgements; for instance, it assumes that conventional caging is neutral, when instead this causes cumulative stress^32^; and any such procedure-based approach risks overlooking practices that modify severity for individual animals (e.g. refinements in technique or analgesic use; animals’ temperaments; how handling styles affect fear of humans; how social buffering and housing quality affect resilience). Furthermore, even texts that include the welcome addition of animal-based welfare indicators (e.g. De Vleeschauwer et al.^33^) still generally restrict these to clinical signs only (ignoring cognitive, physiological, immunological and behavioural signs of negative affect), as well as leaving opaque what makes something a sign of mild versus moderate versus severe impact. Thus as Reiber et al.^7^ summarise, the central problem is ‘we do not yet have . . . a gold standard combination of severity assessment parameters that reflects the actual truth about severity’.

Thinking formally about construct validation could again help by laying out logical, explicit frameworks to advance progress. For example, Test 1 again highlights the value of using data from relevant human patients, here for identifying measurable signs that reflect self-reported mild, moderate or severe reductions in quality of life. Tests 2 and 3 suggest merit in seeking indicators that differentiate between animals exposed to situations ranging from mildly to intensely aversive (Test 2), or from subtly to devastatingly harmful to fitness (Test 3). And Test 4’s pharmacological approach indicates another route: identifying whether affect-rectifying drugs influence indicators even at very low doses (as expected for indicators of mildly negative states) or only when doses are maximally high (as expected for indicators of very severe states).

Even the most careful construct validation will never fully solve the ‘Other Minds’ problem: that subjective experiences are private. However, done well it would add more logical rigour to laboratory animal welfare assessment by grounding this in sound biological principles, and by encouraging underlying assumptions to be made explicit. In turn this should reduce risks of false leads or false null errors, and make benchmarking (e.g. severity classification) more transparent and defensible. It could even increase the translatability of biomedical research to human patients (cf. Krock et al.^34^, Gorman and Davies^35^).
